# Analysis of ovarian reserve markers (AMH, FSH, AFC) in different age strata in IVF/ICSI patients

**Published:** 2016-08

**Authors:** Ensieh Shahrokh Tehraninezhad, Fatemeh Mehrabi, Raheleh Taati, Vahid Kalantar, Elham Aziminekoo, Azam Tarafdari

**Affiliations:** *Vali-e-Asr Reproductive Health Research Center, Department of Obstetrics and Gynecology, Imam Khomeini Complex Hospital, Tehran University of Medical Sciences, Tehran, Iran.*

**Keywords:** *Anti-mullerian hormone*, *Ovarian reserve*, *Follicle stimulating hormone*, *Ovarian follicles*

## Abstract

**Background:**

The predictive roles of follicle stimulating hormone (FSH), anti-mullerian hormone (AMH) and antral follicle count (AFC) as ovarian reserve markers in women with different age groups are not established well.

**Objective:**

This study compares the value of FSH, AMH and AFC at the time of in vitro fertilization (IVF) treatment in different age groups.

**Materials and Methods:**

In this cross-sectional study, 103 women aged 20-43 years candidates for IVF/ICSI cycle were recruited. FSH, AMH and AFC on day 3 of menstrual cycle were measured. The relationship of these measured markers with outcome variables (oocytes number, number of frozen/fresh embryo and chemical and clinical pregnancy) was assessed in different age groups (i.e. 20-32, 33-37 and 38-43 years).

**Results:**

our results show that age was correlated with clinical pregnancy, oocyte count and fresh and frozen embryo (p<0.001). AMH, AFC and FSH were not correlated with clinical or chemical pregnancy at total population or age subgroups except the significant correlation of AFC with clinical pregnancy at 33-37 years old group. AFC was correlated with oocyte count and the number of fresh and frozen embryos in the ages group 20-32 years. In this age group, AMH was correlated with fresh and frozen embryos. AMH, AFC and FSH were correlated with oocyte count and the number of fresh embryos in age group 33-37 years. AMH was correlated with oocyte count and the number of fresh embryos in 38-43 years old group.

**Conclusion:**

We concluded that the age is the superior predictor of IVF outcome and AMH and AFC are variable predicting markers of ovarian reserve in different age groups.

## Introduction

Prevalence of infertility range is 3.5-16.7% in developed countries and 6.9-9.3% in developing countries. It is estimated that overall median prevalence of infertility is 9% worldwide (1). In Iran, the overall prevalence of infertility and the weighted national estimate of primary infertility are 8% and 4.6%, respectively (2).

The increased age of first delivery is the main causes of infertility due to diminished ovarian reserve secondary to aging (3-5). Unexpected problems in becoming pregnant will be observed in many older women due to decreased ovarian reserve (5, 6). While attempts for becoming pregnant at earlier ages is considered as the only and the best effective treatment for diminished ovarian reserve, many researchers studied to identify predictors of ovarian reserve (7). Indeed many tests have been previously introduced to predict in-vitro fertilization (IVF) outcome (8-10). 

Now it is well acknowledged that some named predictors of ovarian reserve markers including serum follicle stimulating hormone (FSH) concentration, antral follicle count (AFC) and serum anti-Mullerian hormone (AMH) concentration can be used as predictors of ovarian responses to gonadotropin stimulation during IVF treatment (11-14). Although, some reports have shown a poor predictive performance of these markers, other reports indicate these predictors can be used accurately for estimation of success rate before initiation of the therapy process (6, 11, 14-16). For example, AMH and ultrasound assessment of the AFC has been discovered to be a valuable clinical predictor of ovarian response to hyperstimulation (17, 18). 

The age-related decrease in female fertility can be most likely attributable to deterioration in quantity and quality of oocytes. As a result of diminished ovarian reserve, the women’s ability to conceive naturally will limit after the age of 40 (8, 19). Although there is assumptions indicating that the relationship between age and ovarian reserve is highly variable and the potential different validity of ovarian reserve markers in women in different age groups remains to be demonstrated (4, 5). 

There are not enough studies assessing the predictive value of these markers in different age strata since some ovarian reserve markers may have different accuracy in different ages. The aim of the current study was to examine the predictive value of AMH, AFC and FSH to predict clinical/chemical pregnancy rate, live birth and ovarian response to controlled ovarian hyper-stimulation (COH) in an IVF cycle in population with different age groups.

## Materials and methods

A total of 103 women with indication for IVF/ICSI treatment were recruited in this cross-sectional study from April 2012 to April 2013. The research was approved by the ethics committee and the Department of Gynecology and Obstetrics of Tehran University of Medical Sciences. Written inform concent was obtained from all participants.

Patients were enrolled from two referral centers. Infertile patients aged 20-43 years candidates for IVF/ICSI were included into the study using convenient sampling method. The exclusion criteria comprised; any history of hypertension (controlled or uncontrolled), previous ovarian surgery, hormonal therapy in the past 6 months, malignancy or exposure to cytotoxic drugs and pelvic radiotherapy, endocrinological disorders including abnormal prolactine, TSH or diabetes and moderate/severe endometriosis. 

**Markers and study outcomes**

FSH and AMH were quantified in the morning of day 3 of the menstrual cycle. The AFC and normal anatomy of pelvic organs were determined by performing transvaginal ultrasonography (TVS). The main outcome measures were the number of retrieved oocytes, chemical/clinical pregnancy rate along with the number of fresh and frozen embryos. The oocyte stands for metaphase II oocytes throughout the manuscript. 

**Stimulation protocol**

Two protocols were applied on study population. In Long protocol GnRh agonist 0.5 mg Buserelin acetate (Superfact, Sanofi, Canada) was administered on day 21 of previous menstrual cycle. Endometrial thickness less than 4 mm indicated down regulation. Then treatment with gonadotropin either Gonal-F (Serono) or Menopur (Ferring) was started. In long fixed and multi-dose GnRh antagonist protocol, we started antagonist (Cetrorelix; Serono) 0.25 mg daily on day 13 of stimulation cycle.

In both protocols different doses of gonadotropins were administered depending on age, presence of PCOS, and/or ovarian responses in prior cycle. Then according to measurements of follicular count by TVS, obtained every two or three days, gonadotropin doses were adjusted. When three follicles larger than 18 mm were observed, 10000 IU of HCG (Pregnyl, Organon) was administered intramuscularly. Thirty six hours later follicles were collected by transvaginal ultrasonography-guide under general anesthesia. 

The number of retrieved oocytes was recorded. After fertilization of oocytes with sperm in the laboratory, embryos were transferred based on patients' age and the numbers of embryos available. All of the patients were administered by Cyclogest suppository (Alpharma, Barnstaple, UK) 400 mg daily until 12 wks of gestation in order to support luteal phase. Patient's serum β HCG were checked 14 days later.

**Statistical analysis**

The potential associations between study predictors (AMH, FSH and AFC) and outcome variables (chemical /clinical pregnancy, oocyte count, frozen and fresh embryo) were determined in different age groups including 20-32, 33-37 and 38-43 years. 

We used Man-Whitney, independent t-test, Spearman's r and Pearson coefficient when appropriate to analyze the differences and correlations. Normality of numeric variables was assessed using Kolmogorov Smirnov test. p˂0.05 were considered statistically significant. All analysis was performed using Statistical Package for the Social Sciences version 20 (SPSS Inc, Chicago, Illinois).

## Results

Of the 103 patients involved in the study, 67 (65%) underwent long GnRH agonist protocol and the remaining 36 participants underwent long fixed and multi-dose GnRH antagonist protocol. The etiology of infertility was recorded as ovarian factor (n=37; 36%), male factor (n=35; 34%), tubal factor (n=13; 13%), endometriosis (n=3; 3%) and unexplained infertile case (n=13; 13%). Two patients were entered IVF procedure for sex selection purposes (2%). 

The cycle was canceled in only one patient secondary to poor response. Also, embryo transfer (ET) was canceled in 4 patients because of ovarian hyperstimulation. From the remained patients, 2 (1.9%) had chemical pregnancy and 31 (31.6%) experienced clinical pregnancy. In total, AMH, AFC or FSH failed to predict clinical and/or chemical pregnancy (p>0.05, Table I). AMH and AFC had significant positive correlations with the number of retrieved oocyte (p=0.001) and fresh or frozen embryo (p=0.001, Table II). While FSH did not correlate with any outcome (Table II).

Age was correlated significantly with the oocyte count (r=0.45), fresh embryo (r=0.45) and frozen embryo (r=0.40, all p<0.001). The age stratified analyses are shown in tables I,II. Considering abnormal distribution of AMH and FSH values, we employed non-parametric tests (i.e. Spearman and Man-Whitney) for the related analyses. For AFC we applied parametric tests. In age group 20-32 years, none of the studied factors predicted clinical and chemical pregnancy. AFC had significant positive correlations with oocyte count and both AMH and AFC levels correlated with fresh or frozen embryo counts (p<0.05); FSH did not correlate with oocyte or embryo number in this age group. No chemical pregnancy occurred in 20-32 and 33-37 years age groups. In age group 33-37 years, only AFC could significantly predict clinical pregnancy (Table I). 

Mean of AFC in women with clinical pregnancy was significantly higher than women without clinical pregnancy (p=0.028). AFC, AMH and FSH levels significantly correlated with oocyte count and fresh embryo count; but none of them showed correlation with the number of frozen embryo (Table II). In age group 38-43 yrs, studied predictor factors did not predict clinical and chemical pregnancy (p>0.05, Table I). AMH but not AFC or FSH levels had significant correlations with oocyte count or fresh embryo number. None of studied factors were correlated with frozen embryo (p>0.05). 

To more investigate the correlation of ovarian reserve markers with clinical pregnancy a binary logistic model was designed with age-groups (categorical) and any marker level. Age was correlated independent of AFC with clinical pregnancy (p=0.034) but AFC was not correlated with clinical pregnancy independent of the effects of age. The interaction effects of FSH and age (p=0.3) as well as AMH and age (p=0.7) on their association with clinical pregnancy were not significant but age was correlated to clinical pregnancy independent of either FSH or AMH (p=0.036 and p=0.047 respectively). With regard to the oocyte count, AMH (beta=0.3, p=0.001) and age (beta=-0.3, p=0.01) were independent predictors of oocyte count. 

Among AFC and age, AFC was the independent predictor (beta=0.6, p=0.001). Among FSH and age, age was the only independent predicting variable (beta=-0.4, p=0.001). To assess the effect of the COH protocol, general linear models were designed with protocol type (i.e. agonist vs. antagonist) as the confounding factor. Protocol had no interaction effect on the association of AMH, AFC or FSH with clinical pregnancy. Also the association of the AFC or AMH with oocyte count remained unchanged and yet significant after the inclusion of the protocol type into the regression model.

**Table I:** The associations between study predictors and pregnancy in different age strata

**Age**		**Clinical pregnancy**				**Chemical pregnancy**			
		**Yes**		**No**		**Yes**		**No**	
		**Mean**	**SD**	**Mean**	**SD**	**Mean**	**SD**	**Mean**	**SD**
20-32 y									
	AMH	4.2	2.9	3.9	2.8	-	-	4	2.8
	AFC	14	5.4	14.5	6.7	-	-	14.3	6.3
	FSH	5.9	2.7	5.8	2.6	-	-	5.8	2.6
33-37 y									
	AMH	1.7	1	2.6	3	-	-	2.1	2.2
	AFC	12.4†	6	6.2	4.9	-	-	9.3	6.2
	FSH	8.1	3.1	9.4	7.3	-	-	8.7	5.5
38-43 y									
	AMH	1.4	0.8	0.9	0.6	0.6	0.1	1	0.6
	AFC	5	7.9	3.9	3.8	4.5	2.1	4	4.6
	FSH	8.2	3.6	7.8	4.5	5.7	1.2	8	4.4
Total									
	AMH	3	2.5	2.6	2.6	0.6	0.1	2.8	2.6
	AFC	12.1	6.6	9.5	7.5	4.5	2.1	10.4	7.3
	FSH	6.9	3.1	7	4.3	5.7	1.2	7	4

Data are presented as means and standard deviations

AMH: anti-mullerian hormone

FSH: follicle stimulating hormone

AFC: antral follicle count

SD: Standard deviation

† indicate significant difference (p<0.05)

**Table II:** The associations between study predictors and Oocyte and embryo counts in different age strata

**Age**		**Oocyte Count** †	**Sig**	**fresh embryo** †	**Sig**	**Frozen embryo** †	**Sig**
20-32 years							
	AMH	0.226	0.103	0.322	0.019	0.302	0.028
	AFC	0.549	0.000	0.579	0.000	0.432	0.001
	FSH	0.109	0.436	0.031	0.826	0.026	0.852
33-37 years							
	AMH	0.838	0.000	0.838	0.000	0.203	0.420
	AFC	0.721	0.001	0.721	0.001	-0.014	0.956
	FSH	-0.587	0.010	-0.587	0.010	-0.427	0.077
38-43 years							
	AMH	0.739	0.000	0.739	0.000	-0.024	0.897
	AFC	0.200	0.273	0.200	0.273	0.104	0.570
	FSH	-0.081	0.659	-0.081	0.659	0.220	0.227
All							
	AMH	0.688	0.000	0.726	0.000	0.434	0.000
	AFC	0.709	0.000	0.731	0.000	0.460	0.000
	FSH	-0.127	0.203	-0.161	0.104	-0.096	0.337

Sig: p˂0.05

† Data are coefficient of correlation and p-values.

## Discussion

The main reasons of infertility are ovarian factors which are closely associated with the age of patients. These factors provide an estimation of ovarian functional reserve. It has been shown that age and FSH are important predictive factors for IVF outcome (20). Also predictive value of AMH and AFC has been studied previously with controversial viewpoints (6, 15, 21, 22). 

Above all, the ovarian reserve and its biomarkers associate with age. Barbakadze and coworkers previously studied the association of FSH, AMH and AFC with age (23). AFC and AMH decreased and FSH increased with age increments. They concluded that AMH was a more reliable biomarker of ovarian reserve compared to FSH and in addition the combination of AMH and AFC was superior. In another large scale study it was confirmed that in both infertile and fertile women AMH decreases with increase in aging from 24-50 years (24). Unlike the documented association of age and ovarian reserve factors ovarian response to stimulation in different age groups is not well studied. The ovarian response may vary in different ages and the knowledge of the pattern of variations may assist decision making before and during the IVF procedure.

In the current study the associations of ovarian biomarkers with outcomes of IVF in different age groups were studied. In general we found that age was the superior variable to predict clinical pregnancy. Furthermore the AFC and AMH levels predicted oocyte count in different age groups independent of the age of participants. In different age strata the correlations were somehow different; we assume the differences probably reflect the different study power in different age groups rather than real age dependent variability. In a recent study, value of AFC, FSH and AMH were assessed for prediction of oocyte count in antagonist IVF cycles. All 3 biomarkers successfully predicted the outcome but the AFC was more accurate (25). In the current study, assessing the significance of age on this association, we showed that the IVF using either agonist or antagonist protocol had no interaction on the association of the predictors with outcomes. 

We did not determine the predictive value of some ovarian reserve markers in certain age groups secondary to insufficient sample size. The effects of these markers may be sought in future studies with larger sample size. The future studies may also include further factors which may influence the results of the IVF cycles not assessed in the current study including etiologies of the infertility and the use of supplementary medications and antioxidants.

## Conclusion

The main predicting factor for ovarian reserve of IVF candidates in our study was the age of the participants. In different age groups measuring AFC and AMH levels assist prediction of outcome variables including oocytes number, frozen\fresh embryo, chemical and clinical pregnancy. In current study, ovarian reserve markers have shown different predictive ability in different age groups.

## note

***This article extracted from M.D. thesis. (Raheleh Taati)***

## References

[1] Boivin J, Bunting L, Collins JA, Nygren KG (2007). International estimates of infertility prevalence and treatment-seeking: potential need and demand for infertility medical care. Hum Reprod.

[2] Safarinejad MR (2008). Infertility among couples in a population‐based study in Iran: prevalence and associated risk factors. Int J Androl.

[3] Malizia BA, Hacker MR, Penzias AS (2009). Cumulative live-birth rates after in vitro fertilization. New Eng J Med.

[4] Nardo LG, Gelbaya TA, Wilkinson H, Roberts SA, Yates A, Pemberton P (2009). Circulating basal anti-Müllerian hormone levels as predictor of ovarian response in women undergoing ovarian stimulation for in vitro fertilization. Fertil Steril.

[5] Te Velde ER, Pearson PL (2002). The variability of female reproductive ageing. Hum Reprod Update.

[6] BroerSL, Mol BW, Hendriks D, Broekmans FJ (2009). The role of antimullerian hormone in prediction of outcome after IVF: comparison with the antral follicle count. Fertil Steril.

[7] Tremellen KP, Kolo M, Gilmore A, Lekamge DN (2005). Anti-mullerian hormone as a marker of ovarian reserve. Aust N Z J Obstet Gynaecol.

[8] Mutlu MF, Erdem M, Erdem A, Yildiz S, Mutlu I, Arisoy O (2013). Antral follicle count determines poor ovarian response better than anti-Müllerian hormone but age is the only predictor for live birth in in vitro fertilization cycles. J Assist Reprod Genet.

[9] Lekamge DN, Barry M, Kolo M, Lane M, Gilchrist RB, Tremellen KP (2007). Anti-Müllerian hormone as a predictor of IVF outcome. Reprod Biomed Online.

[10] Broer SL, Dólleman M, Opmeer BC, Fauser BC, Mol BW, Broekmans FJ (2011). AMH and AFC as predictors of excessive response in controlled ovarian hyperstimulation: a meta-analysis. Hum Reprod Update.

[11] Li HW, Lee VC, Lau EY, Yeung WS, Ho PC, Ng EH (2013). Role of baseline antral follicle count and anti-Mullerian hormone in prediction of cumulative live birth in the first in vitro fertilisation cycle: a retrospective cohort analysis. PLoS One.

[12] Eldar-Geva T, Ben-Chetrit A, Spitz IM, Rabinowitz R, Markowitz E, Mimoni T (2005). Dynamic assays of inhibin B, anti-Mullerian hormone and estradiol following FSH stimulation and ovarian ultrasonography as predictors of IVF outcome. Hum Reprod.

[13] Broekmans FJ, Kwee J, Hendriks DJ, Mol BW, Lambalk CB (2006). A systematic review of tests predicting ovarian reserve and IVF outcome. Hum Reprod Update.

[14] Nelson SM, Yates RW, Fleming R (2007). Serum anti-Müllerian hormone and FSH: prediction of live birth and extremes of response in stimulated cycles-implications for individualization of therapy. Hum Reprod.

[15] Broer SL, Mol BW, Dólleman M, Fauser BC, Broekmans FJ (2010). The role of anti-Müllerian hormone assessment in assisted reproductive technology outcome. Curr Opin Obstet Gynecol.

[16] Tarlatzis BC, Zepiridis L, Grimbizis G, Bontis J (2003). Clinical management of low ovarian response to stimulation for IVF: a systematic review. Hum Reprod Update.

[17] Fallat ME, Siow Y, Marra M, Cook C, Carrillo A (1997). Müllerian-inhibiting substance in follicular fluid and serum: a comparison of patients with tubal factor infertility, polycystic ovary syndrome, and endometriosis. Fertil Steril.

[18] Ng EH, Tang OS, Ho PC (2000). The significance of the number of antral follicles prior to stimulation in predicting ovarian responses in an IVF programme. Hum Reprod.

[19] Faddy MJ, Gosden RG, Gougeon A, Richardson SJ, Nelson JF (1992). Accelerated disappearance of ovarian follicles in mid-life: implications for forecasting menopause. Hum Reprod.

[20] Taylor R (1990). Interpretation of the correlation coefficient: a basic review. J Diagnost Med Sonography.

[21] Eftekhar M, Rahmani E, Pourmasum S (2014). Evaluation of clinical factors influencing pregnancy rate in frozen embryo transfer. Iran J Reprod Med.

[22] Aflatoonian A, Oskouian H, Ahmadi S, Oskouian L (2009). Prediction of high ovarian response to controlled ovarian hyperstimulation: anti-Müllerian hormone versus small antral follicle count (2-6 mm). J Assist Reprod Genet.

[23] Barbakadze L, Kristesashvili J, Khonelidze N, Tsagareishvili G (2015). The Correlations of Anti-Mullerian Hormone, Follicle-Stimulating Hormone and Antral Follicle Count in Different Age Groups of Infertile Women. Int J Fertil Steril.

[24] Seifer DB, Baker VL, Leader B (2011). Age-specific serum anti-Müllerian hormone values for 17,120 women presenting to fertility centers within the United States. Fertil Steril.

[25] Tsakos E, Tolikas A, Daniilidis A, Asimakopoulos B (2014). Predictive value of anti-müllerian hormone, follicle-stimulating hormone and antral follicle count on the outcome of ovarian stimulation in women following GnRH-antagonist protocol for IVF/ET. Arch Gynecol Obstet.

